# Complete Genome Sequence of *Pseudomonas aeruginosa* MTB-1, Isolated from a Microbial Community Enriched by the Technical Formulation of Hexachlorocyclohexane

**DOI:** 10.1128/genomeA.01130-13

**Published:** 2014-01-23

**Authors:** Yoshiyuki Ohtsubo, Takuya Sato, Kouhei Kishida, Michro Tabata, Yoshitoshi Ogura, Tetsuya Hayashi, Masataka Tsuda, Yuji Nagata

**Affiliations:** a Department of Environmental Life Sciences, Graduate School of Life Sciences, Tohoku University, Sendai, Japan; b Division of Genomics and Bioenvironmental Science, Frontier Science Research Center, University of Miyazaki, Miyazaki, Japan

## Abstract

*Pseudomonas aeruginosa* MTB-1 does not degrade gamma-hexachlorocyclohexane (γ-HCH), but this bacterium persistently coexists with a γ-HCH-degrading strain, *Sphingomonas* sp. MM-1, in a microbial community enriched by the technical formulation of HCH. Here we report the complete MTB-1 genome sequence, with a 6.6-Mb circular chromosome.

## GENOME ANNOUNCEMENT

Gamma-hexachlorocylohexane (γ-HCH) (also called gamma-benzenehexachloride or lindane) is a chlorinated organic insecticide that has caused serious environmental problems due to its toxicity and long persistence in upland soils (1, 2). The technical formulation of HCH (t-HCH) mainly consists of an insecticidal γ-isomer and noninsecticidal α-, β-, and δ-isomers. Our previous use of a microbial community enriched by t-HCH from t-HCH-contaminated soil in India (5) led to the successful isolation of a γ-HCH-degrading bacterium, *Sphingomonas* sp. strain MM-1 (3, 4), and a non-γ-HCH-degrading bacterium, *Pseudomonas aeruginosa* MTB-1 (3). The strains MM-1 and MTB-1 have been deposited in the Japan Collection of Microorganisms (JCM) under the accession numbers JCM 19685 and JCM 19686, respectively.

The MTB-1 genome was sequenced using 454 GS FLX+ (Roche) and MiSeq (Illumina) systems. A fragment library and a paired-end library were constructed for the 454 sequencing, and 229,833 reads (179 Mb) and 44,402 reads (19 Mb) were obtained, respectively, while the 151-bp paired-end sequencing with Illumina generated 3,862,256 reads (578 Mb). The reads obtained by the two systems were assembled using Newbler version 2.8 (Roche), which produced 45 contigs and a single scaffold. The finishing was facilitated using the two computer programs GenoFinisher and AceFileViewer (6) (http://www.ige.tohoku.ac.jp/joho/gf_e/). The scaffold consisted of 15 nonrepeat contigs, and all of the 15 gaps, including those located between the last and the first contigs, were closed by *in silico* analyses, in which the DNA sequences of each copy of repeats were precisely determined. The finished sequence was confirmed by FinishChecker, an accessory tool of GenoFinisher.

The sequence was annotated by the NCBI Prokaryotic Genomes Automatic Annotation Pipeline (PGAAP), and the resulting annotation was subjected to manual curation using the annotation support tool of GenomeMatcher (7). In the curation, by referencing annotation data obtained from the Microbial Genome Annotation Pipeline (http://www.migap.org/), we corrected the start codon positions and added several genes that were missing in the PGAAP annotation.

The complete sequence of the MTB-1 genome consisted of one circular chromosome with a size of 6,580,038 bp. It carried four copies of rRNA operons, 64 tRNA genes, and 6,078 protein-encoding genes. The six *lin* genes specific for the conversion of γ-HCH to β-ketoadipate (*linA* to *linF*) (8) were not found in the MTB-1 genome. A detailed comparison of the MTB-1 genome with the genomes of other *P. aeruginosa* strains may provide us with some insights into the reason(s) why MTB-1 persistently coproliferated with the γ-HCH degrader MM-1 in the liquid culture enriched by t-HCH.

### Nucleotide sequence accession number.

The genome sequence of *P. aeruginosa* MTB-1 has been deposited in GenBank under the accession number CP006853.
